# The role of chrysanthemum phytochemicals in neural tube development: a narrative review of underlying mechanisms

**DOI:** 10.3389/fcell.2026.1804458

**Published:** 2026-05-21

**Authors:** Jiaojiao Gao, Yage Dang, Yajing Chang, Yurong Liu, Yuqing Sun, Bei Zhao, Pinglian Sun, Xiuwei Wang, Jing Wang, Li Zhang

**Affiliations:** 1 Department of Biological and Food Engineering, Lyuliang University, Lvliang, Shanxi, China; 2 Department of Neurology, The First Hospital of Shanxi Medical University, Taiyuan, Shanxi, China; 3 Department of Biochemistry and Molecular Biology, Shanxi Medical University, Taiyuan, Shanxi, China; 4 Department of Hepatobiliary Surgery and Liver Transplant Center, The First Hospital of Shanxi Medical University, Taiyuan, Shanxi, China; 5 Department of Pharmacy, Shanxi Second People’s Hospital, Taiyuan, Shanxi, China; 6 Department of Intensive Care Unit, Lvliang People’s Hospital, Lvliang, China; 7 Beijing Municipal Key Laboratory of Child Development and Nutriomics, Capital Institute of Pediatrics, Beijing, China

**Keywords:** Chrysanthemum morifolium, dual bioactivity, molecular mechanisms, neural tube defects, neurodevelopment, toxicological evaluation

## Abstract

**Background:**

Neural tube defects (NTDs) have long been attributed to genetic and hereditary factors, but emerging evidence highlights the significant role of pharmacological and environmental agents. The global integration of traditional medicine systems, including Traditional Chinese Medicine, has introduced diverse bioactive compounds with potential to influence neurodevelopmental processes.

**Purpose:**

This narrative review aims to comprehensively summarize and critically evaluate the current evidence on the bioactivity of chrysanthemum (Chrysanthemum morifolium and related species) extracts and their constituents in the context of neural tube development, with a focus on elucidating underlying molecular mechanisms to inform potential therapeutic applications and toxicological risks.

**Study Design:**

We performed a narrative review of chrysanthemum compounds in neural development, focusing on neural tube formation. PubMed, Web of Science, and ScienceDirect were searched from January 1985 to March 2026 using terms related to chrysanthemum (e.g., Chrysanthemum, extract, phytochemicals), neurodevelopmental outcomes (neural tube defects, neurulation), and mechanisms (inflammation, oxidative stress, apoptosis). Reference lists were manually screened. No formal bias assessment or quantitative synthesis was performed; instead, findings were qualitatively synthesized to present the dual role of chrysanthemum.

**Results:**

Chrysanthemum exhibits context-dependent duality in neural tube development. Beneficial effects are mediated by chlorogenic acid, Eri-7-Glc, and flavonoids via NF-κB, Nrf2/HO-1, and Wnt/β-catenin pathways, reducing oxidative stress and supporting neurulation. Conversely, natural pyrethrins induce oxidative stress and DNA damage; high-dose flavonoids act as pro-oxidants and endocrine disruptors, and readily cross the placenta, raising fetal concerns. The convergence on caspase-8 suggests a double-edged sword effect. However, direct evidence in mammalian embryos, pregnancy pharmacokinetics, and dose-response data are lacking.

**Conclusion:**

Chrysanthemum shows context-dependent dual effects on neurodevelopment, underscoring the need for rigorous mechanistic and toxicological studies to guide its safe use, particularly in pregnancy.

## 1 Introduction

Chrysanthemum, a traditional Chinese medicinal herb with a long history of use, has been widely applied in various therapeutic contexts. The plant’s diverse bioactive constituents and broad pharmacological activities render it a subject of significant research interest in both traditional and modern medicine (Eisa et al., 2022). With rising living standards and growing emphasis on healthy dietary practices, chrysanthemum has gained increasing attention as a functional food ingredient. The plant contains a wide range of active compounds, including polysaccharides, flavonoids, volatile oils, terpenoids, phenylpropanoids, and other polyphenolic substances (Ma et al., 2016). Pharmacologically, chrysanthemum exhibits multiple beneficial effects, such as enhancing myocardial contractility, antitumor, anti-inflammatory, antioxidant, antihypertensive, and lipid-lowering activities, along with improvements in immune and digestive functions (Jiang et al., 2021). These multifaceted properties therefore support the broad integration of chrysanthemum into pharmaceutical agents, nutritional supplements, and daily health products, underscoring its significance in both traditional and modern healthcare contexts.

However, the pharmacological profile of chrysanthemum is complex and context-dependent, exhibiting dualistic effects in biological systems. For instance, certain chrysanthemum extracts may promote aspects of neurodevelopment while simultaneously posing a risk of inducing neural tube defects (NTDs) and other neurodevelopmental disorders such as Parkinson’s disease (PD). Similarly, some chrysanthemum constituents display both anti-inflammatory and pro-inflammatory activities, with the latter potentially contributing to adverse reactions such as allergic dermatitis.

NTDs represent a class of congenital malformations resulting from incomplete closure of the neural tube during embryogenesis, primarily presenting as spina bifida, anencephaly, and encephalocele. Proper neural tube formation relies on tightly regulated proliferation and differentiation of neural epithelial cells. These processes are modulated by a network of transcriptional regulators, growth factors, extracellular matrix proteins, and associated genes. Disruption in the balance between neural epithelial cell proliferation and apoptosis can compromise neural tube development and lead to NTDs. This narrative review summarizes the active components, pharmacological properties, and mechanisms of action of chrysanthemum, focusing on its dual role in neural tube development.

## 2 Phytochemical composition and pharmacology of chrysanthemum species

Chrysanthemum, a perennial plant of the Asteraceae family, has a long history of use in China, particularly in the form of tea chrysanthemum. Its main bioactive constituents include flavonoids, volatile oils (VOs), phenylpropanoids, polyphenols, terpenoids, and amino acids. Flavonoids and phenylpropanoids are considered the primary pharmacologically active components. Notably, flavonoid compounds such as luteoloside, quercetin, and apigenin are among the most abundant (Ouyang et al., 2022). Phenylpropanoids, including simple forms (e.g., caffeic acid, chlorogenic acid) and coumarins (e.g., imperatorin), contribute significantly to chrysanthemum antipyretic, analgesic, and antioxidant effects. VOs represent another major class of chemical components in chrysanthemum, demonstrating a broad spectrum of pharmacological activities such as antibacterial, antioxidant, antitumor, anti-inflammatory, and sedative-hypnotic properties (Zhang J. et al., 2024). Analysis of chrysanthemum VOs has identified up to 26 components, including eucalyptol, camphor, and borneol (Kuang et al., 2018). The rich and diverse profile of these bioactive compounds establishes chrysanthemum as a complex botanical agent with the potential to modulate intricate biological processes such as embryonic development.

## 3 Determinants of neural tube development

### 3.1 Neural tube formation: morphogenesis and critical windows

NTDs are multifactorial disorders arising from the interplay of genetic susceptibility and environmental exposures during critical windows of neural tube formation (Copp et al., 2013). In humans, around embryonic day 16 (E16), ectodermal cells near the midline invaginate and fuse to form the neural tube. The notochord induces thickening of the adjacent ectoderm to form the neural plate, whose lateral edges elevate to form neural crests while the central portion sinks as the neural groove. The crests converge and fuse, creating a hollow tube with anterior and posterior neuropores. The anterior neuropore closes by day 25, the posterior by day 27. Failure of anterior closure causes anencephaly; failure of posterior closure causes spina bifida (Greene and Copp, 2014).

### 3.2 Inflammatory, metabolic, and genetic contributors to NTDs

Inflammation is a recognized modulator of neural tube development. Low-grade chronic inflammation disrupts folate metabolism, elevates homocysteine, and impairs neural tube closure (van der Windt et al., 2021). Folate deficiency reduces S-adenosylmethionine (SAM) synthesis, leading to hyperhomocysteinemia, which activates pro-inflammatory transcription factors and exacerbates inflammation (Kwon et al., 2014; Duthie et al., 2015). Our previous studies showed that SAM metabolic dysfunction induces oxidative stress, impairs lysosomal degradation and mitophagy, downregulates Pax3 expression (suppressing proliferation and promoting apoptosis), and modulates METTL3-mediated m6A RNA modification, thereby suppressing Wnt/β-catenin signaling and contributing to NTDs (Zhang et al., 2021a; Zhang L. et al., 2024; Zhang et al., 2020). Epidemiological studies have established maternal fever during pregnancy as a significant risk factor for NTDs. Maternal fever, infections, and inflammatory markers such as VCAM-1 and MDA are also linked to NTD risk (Wilde et al., 2014; Dreier et al., 2014; Botto et al., 2002; Acs et al., 2005). Periconceptional folic acid supplementation may attenuate this risk.

In addition to genetic susceptibility, multiple environmental and physiological factors—such as nutritional imbalances, folic acid deficiency, hyperthermia, radiation, organic compound exposure, and maternal infections-can disrupt neural tube closure. Notably, Chrysanthemum indicum extract (CISCFE) activates the Wnt/β-catenin pathway (Nie et al., 2021), which is a key regulator of NTDs. Conversely, chrysanthemum methanol extract (CME) inhibits MAPK/c-fos activation (Jang et al., 2021), a pathway linked to NTD susceptibility. Therefore, we speculate that chrysanthemum may influence NTD pathogenesis by modulating these pathways, although the specific mechanisms—particularly the context-dependent duality of Wnt/β-catenin, MAPK/c-fos, and ERK signaling—remain largely unknown. Future studies using targeted gene knockout, pathway-specific inhibitors, and animal models are needed to clarify these mechanisms. This delicate balance between genetic programs and external factors makes the developing neural tube vulnerable to modulation by exogenous bioactive compounds, including those from medicinal plants.

## 4 Effects of chrysanthemum bioactives on neural tube development

### 4.1 Chrysanthemum compounds act as a double-edged sword bridging antitumor apoptosis and neural tube development

As introduced in the previous section, chrysanthemum extracts have been shown to modulate key signaling pathways such as Wnt/β-catenin and MAPK/c-fos, which are implicated in NTD pathogenesis. Here we further explore the dual role of chrysanthemum compounds, focusing on the convergence of antitumor apoptosis and neural tube development. Chrysanthemum bioactive exhibit potent antitumor properties, primarily through inducing apoptosis in cancer cells. A key mechanism involves sesquiterpene lactone compounds (e.g., CA), which trigger caspase-8-dependent apoptosis and upregulate death receptor DR5 via JNK-mediated pathways, highlighting their potential in cancer therapy (Salazar-Gómez et al., 2020). Studies have shown that the effective group with antitumor activity in chrysanthemum is the sesquiterpene lactone compound with an α-methylene-γ-butyrolactone ring, which has strong antitumor activity (Luo et al., 2019). Wild chrysanthemum’s petroleum ether/ethyl acetate extracts exhibit antitumor effects via anti-proliferative, anti-migratory and pro-apoptotic mechanisms (Li et al., 2020). Intriguingly, the caspase-8 pathway, pivotal in the antitumor action of Chrysanthemum, is also fundamentally involved in embryonic neural tube development. Gene-targeting studies in mice have demonstrated that caspase-8 deficiency leads to severe neural tube and cardiac defects, resulting in embryonic lethality by E11.5, underscoring its indispensable role in embryogenesis (Sakamaki et al., 2002). Conversely, elevated caspase-8 activity has been shown to confer a protective effect against spina bifida in model systems (Wang et al., 2017), This convergence on caspase-8 suggests a concentration-dependent dual role for chrysanthemum compounds like CA. At defined concentrations or in specific contexts, they may protect neural tube development by facilitating essential caspase-8-mediated survival or differentiation signals, at higher levels or in distinct cellular environments. However, the same pro-apoptotic mechanism could disturb the delicate equilibrium of neural progenitor cell proliferation and apoptosis, potentially leading to neurodevelopmental toxicity. Thus, chrysanthemum’s impact on neural tube morphogenesis may be critically dependent on its modulation of this shared molecular node, linking the oncological pharmacology directly to developmental biology.

### 4.2 Speculation on the signaling pathway mechanism of chrysanthemum involvement in NTD pathogenesis

Studies have established that planar cell polarity (PCP) signaling and convergent extension movements are critical for neural tube closure, and that disruption of these processes leads to NTDs (Nychyk et al., 2022). Notably, fermentation of Chrysanthemum indicum with Lactococcus lactis activates the Shh signaling pathway, which is a key regulator of NTD pathogenesis (Cho et al., 2024; Brooks et al., 2020). Moreover, numerous studies have reported that the Shh signaling pathway is the most important regulatory pathway in the pathogenesis of NTDs (Litingtung and Chiang, 2000; Brooks et al., 2020). During neural development, neural crest cells that generate the peripheral nervous system, and definitive RP cells of the central nervous system, are sequentially formed in the same anlagen (Rekler and Kalcheim, 2021). The development of the dorsal neural tube embodies fundamental processes in developmental biology: regulation of cell proliferation, cell movements and cell elongation, epithelial-mesenchymal transition, lineage decisions, and the interrelationships among them. Additionally, the formation of neural crest and roof plate involves multiple pathways including BMP, Wnt, Notch, and SHH (Zhang et al., 2021a; Wang Y. et al., 2023; Chen et al., 2021; Li et al., 2023; Katunzi et al., 2025; Zhang et al., 2025).

Beyond direct developmental signaling, inflammatory cytokines represent another layer of regulation that can disrupt neurulation. Cytokines such as TNF-α, IL-6, and IL-1β can directly interfere with neurulation through multiple interconnected mechanisms. First, they activate NF-κB signaling in neuroepithelial cells, which suppresses the expression of PCP genes such as Vangl2 and Celsr1 that are essential for neural tube closure. Second, they induce apoptosis in the neuroepithelium via caspase-3 activation, leading to failure of neural fold elevation and fusion. Third, they disrupt the actin cytoskeleton and cell-cell adhesion molecules including N-cadherin and β-catenin, which are critical for the dynamic shape changes required during neural fold apposition. Finally, they can cross-talk with key developmental pathways such as Wnt/β-catenin, Notch, and SHH, thereby altering neural progenitor proliferation and patterning (Huang et al., 2025; Kim et al., 2025; Zhang et al., 2020; Zhang et al., 2021a).

Based on these observations, chrysanthemum may theoretically influence NTDs by modulating these developmental pathways, although direct experimental validation is still lacking. Emerging evidence also suggests that chrysanthemum constituents, particularly flavonoids and terpenoids, can interfere with GABAergic signaling—a pathway essential for neural tube closure and early neurogenesis (Bhargava and Tyagi, 2014; Briner, 2001; Furukawa et al., 2019). Flavonoids and terpenoids, two major classes of bioactive compounds in chrysanthemum, have been implicated in the modulation of GABAergic transmission (Bavarsad et al., 2023; Min et al., 2021). Certain flavonoids act as positive allosteric modulators of GABAA receptors, while terpenoids may affect GABA metabolism via GAD or GABA-T enzymes (Nguyen et al., 2024). However, direct evidence linking chrysanthemum-derived compounds to specific GABAergic mechanisms in neurulation remains scarce. Further studies are needed to elucidate their molecular targets and structure-activity relationships, which could eventually position these compounds as candidates for NTD prevention or treatment.

### 4.3 Potential mechanism of chrysanthemum in reducing the risk of NTDs by improving obesity-related metabolic disorders

Chrysanthemum is rich in polyphenols and flavonoids with lipid-lowering, anti-inflammatory, and hypotensive properties (Kato et al., 1987). Maternal obesity is a well-established risk factor for NTDs, as it alters metabolic and placental function. Cardiometabolic health, particularly obesity and hyperlipidemia, is a well-established risk factor for hypertension and is increasingly linked to adverse neurodevelopmental outcomes (Mohseni et al., 2024; Bozkurt et al., 2016). Obesity is associated with neuroinflammation, suppressed neurogenesis, and increased risks of neurodegenerative and neurodevelopmental disorders (Morys et al., 2024; Liu et al., 2024; Morys et al., 2023; Bozkurt et al., 2016). During pregnancy, maternal obesity alters metabolic and placental function, raising the risk of NTDs and other developmental anomalies (King, 2006). Studies have reported that compared with normal-weight pregnant women, obese pregnant women have a significantly increased risk of NTDs (Vena et al., 2022). Chinese studies have replicated these findings, with evidence suggesting that maternal underweight status may elevate the risk of anencephaly (Zhang et al., 2021b), though this association was not observed in Shanxi Province cohorts. While some regional variations exist, evidence from Chinese cohorts also supports an association between maternal obesity and increased NTD risk (Li et al., 2015). Given these observations, we speculate that chrysanthemum bioactive compounds may indirectly reduce the risk of obesity-associated NTDs by improving cardiovascular and metabolic parameters, thereby supporting a healthier intrauterine environment and promoting normal neural tube development.

### 4.4 Immunomodulatory properties of chrysanthemum polysaccharides and their potential role in neural tube development

Emerging evidence suggests that immune function plays a direct and active role in neural development. Chrysanthemum polysaccharides have been shown to modulate immune responses by enhancing cellular phagocytosis, promoting lymphocyte proliferation, and upregulating the release of cytokines such as IL-2, IFN-γ, and nitric oxide (NO), indicating their potential as immunostimulatory agents with low cytotoxicity (Wang et al., 2022). Similarly, Jeong et al. reported that chrysanthemum polysaccharides strengthen antigen-specific T cell responses and enhance Th1-mediated immunity, further supporting their immunomodulatory function (Pingili et al., 2019). Notably, immune dysfunction has been associated with an increased risk of NTDs (Wallingford et al., 2013). Recent studies indicate that immune-related proteins are highly expressed during neural tube formation in animal models, suggesting that immune factors may directly participate in neurodevelopmental processes (Gendron et al., 1991; Lomaga et al., 2000; Undas et al., 2004). Data confirmed that small extracellular vesicles derived from hair follicle neural crest stem cells deliver miR-21-5p to inhibit Smad7 expression, activate the TGF-β/Smad signaling pathway, and upregulate hyaluronan synthase 2, thereby enhancing the proliferation, migration, and tight junction protein expression of perineurial cells, and promoting the repair and functional recovery of peripheral nerve defects (Huo et al., 2026). Based on these observations, we hypothesize that Chrysanthemum polysaccharides may indirectly support normal neural tube development through their immunomodulatory effects. Specifically, by enhancing immune homeostasis and cytokine signaling, Chrysanthemum helps maintain an optimal microenvironment for neural morphogenesis, thereby reducing the risk of immune-related disruptions in neural tube closure.

### 4.5 Chrysanthemum bioactive compounds modulate inflammatory pathways to influence neural tube closure a mechanistic hypothesis

Chrysanthemum also contains several anti-inflammatory compounds, mainly including chlorogenic acid, luteolin-7-O-glucoside, and linarin. Chlorogenic acid reduces levels of pro-inflammatory mediators such as IL-2, TNF-α, and MDA, demonstrating efficacy against oxidative arthritis (Xia et al., 2019). Accumulating evidence indicates that oxidative stress activates the NF-κB signaling pathway, triggering the release of pro-inflammatory cytokines and subsequent neuroinflammatory and hepatotoxic damage (Luo and Song, 2021). Similarly, quercetin, a major chrysanthemum flavonoid, inhibits macrophage activation and suppresses NF-κB signaling, thereby lowering the expression of TNF-α, IL-1β, and iNOS (Comalada et al., 2005). Other flavonoids such as handelin and eriodictyol-7-O-β-D-glucoside (Eri-7-Glc) alleviate inflammation by blocking the NF-κB signaling pathway and further modulating the ERK/MAPK pathway (Pyee et al., 2014). These compounds collectively highlight chrysanthemum’s capacity to mitigate inflammatory signaling through multiple molecular targets.

Based on all the above findings, we hypothesize that chrysanthemum’s anti-inflammatory components may support neural tube development through several interrelated mechanisms: (1) Chlorogenic acid and Eri-7-Glc reduce oxidative stress markers like MDA, potentially preserving cellular integrity during neurulation; (2) These compounds may help maintain folate metabolism and CIC function, supporting methylation processes essential for neural tube closure; (3) By downregulating TNF-α and VCAM-1, chrysanthemum bioactives could attenuate neuroinflammatory responses that disrupt morphogenesis; (4) Modulation of nitric oxide (NO) pathways—critical for neural tube closure—may be indirectly supported through anti-inflammatory and antioxidant effects.

However, chrysanthemum exhibits context-dependent bioactivity, and its anti-inflammatory or pro-inflammatory effects depend on factors such as compound type, concentration, cell type, and microenvironment (Ljuba et al., 2022; Kanerva et al., 2001). Although most evidence supports its anti-inflammatory properties, certain components (e.g., some sesquiterpene lactones and flavonoids) may trigger immune responses, including allergic contact dermatitis mediated by T-cell-driven hypersensitivity. Therefore, evaluating chrysanthemum’s impact on developmental inflammation requires consideration of compound specificity, dose, exposure timing, and host immune status. Future studies should systematically screen the immunological activities of individual chrysanthemum compounds and define safe and effective concentration windows using neural development models, thereby providing a basis for rational application in NTD intervention.

### 4.6 Antioxidant and neuroprotective effects of chrysanthemum extracts in oxidative stress-related models

Chrysanthemum extracts exhibit potent antioxidant activity. Wild chrysanthemum extract enhances superoxide dismutase (SOD) and glutathione peroxidase (GPx) activities while reducing MDA levels in hypertensive rats and aging mice (Wang et al., 2021). The yellow pigment from wild chrysanthemum shows strong free radical scavenging (100% DPPH elimination at 1 mg/mL). Ethanol extract of C. boreale (BZE) protects against APAP-induced liver injury by reducing ROS and elevating SOD/GSH. Chrysanthemum extract also improves survival in *Drosophila melanogaster* exposed to paraquat and hydrogen peroxide (Paulsen and Andersen, 2020). In aging mouse models, chrysanthemum extract significantly upregulates oxidase activity in the skin, organs, and brain, reduces MDA content, and attenuates histological damage in liver and skin tissues, demonstrating antioxidant and anti-aging effects. Similarly, beyond its hepatoprotective effects, Chrysanthemum boreale has also been shown to confer neuroprotection against oxidative stress in cellular models.

Methanol extract (CBME) protects SH-SY5Y cells from oxidative damage via MAPK pathways, while root extract (CRE) shows neuroprotection in a PD mouse model by enhancing SOD/GPx and reducing brain MDA (Song et al., 2022). These findings collectively support the antioxidant and neuroprotective potential of chrysanthemum. However, certain chrysanthemum preparations have been associated with sex-dependent behavioral impairments, though whether oxidative stress mediates these effects remains unclear. The potential link between chrysanthemum exposure and NTDs is speculative and requires direct experimental testing.

### 4.7 Prenatal exposure to chrysanthemum compounds: pharmacokinetic gaps, pro-oxidant toxicity, and the NTD hypothesis

While Section 4.6 discussed the antioxidant effects of chrysanthemum extracts, certain constituents, as detailed below, exhibit pro-oxidant toxicity. To date, no direct studies have examined the absorption, distribution, metabolism, and excretion of chrysanthemum-derived compounds—specifically pyrethrins and flavonoids—during pregnancy. Pregnancy induces profound physiological changes (e.g., increased plasma volume, decreased albumin, enhanced hepatic metabolism, and increased renal blood flow) that can alter xenobiotic pharmacokinetics (Morgan, 1997), yet empirical data on chrysanthemum constituents are lacking. Regarding placental transfer, pyrethrins may cross the placenta by passive diffusion, while flavonoids are known to readily cross the placenta (Skibola and Smith, 2000). However, the extent of transfer, interaction with placental transporters, and fetal accumulation remain unknown. At high doses, flavonoids can act as mutagens, pro-oxidants, and inhibitors of hormone-metabolizing enzymes, potentially outweighing their beneficial effects. Because flavonoids easily cross the placenta, the unborn fetus may be particularly at risk.

In addition to the pharmacokinetic gaps described above, specific chrysanthemum constituents directly promote oxidative damage. For instance, natural pyrethrins have been shown to induce oxidative stress in human hepatocytes via the Nrf-2 signaling pathway (Yang et al., 2024). Consistently, pyrethrins trigger oxidative stress, DNA damage, and impaired DNA repair in HepG2 cells, and induce hepatotoxicity and lipid metabolism dysregulation in zebrafish (Lu et al., 2022). In neuronal SH-SY5Y cells, pyrethrin exposure leads to caspase-3 activation, calcium overload, ATP depletion, mitochondrial membrane potential collapse, elevated ROS, altered SOD/CAT/GPx activities, and increased MDA levels—collectively indicating oxidative stress-mediated DNA damage and apoptosis (Wang et al., 2024). Furthermore, in zebrafish embryos, pyrethrins cause axial deformities, disrupt lipid metabolism, and dysregulate key neurodevelopmental and PD-associated genes, highlighting their potential to interfere with fundamental developmental and neurological processes (Wang et al., 2024). However, it is important to interpret these toxicological findings in the context of realistic exposure levels. Most studies on pyrethrins use concentrations considerably higher than those encountered through normal dietary or herbal tea consumption (Proudfoot, 2005). In such high-dose settings, carboxyesterase inhibitors can enhance pyrethroid toxicity (Skibola and Smith, 2000). Moreover, epidemiological evidence indicates that prenatal exposure to elevated levels of pyrethroids and their mixtures is associated with reduced Bayley scores in children (Wang A. et al., 2023). Given these findings, we explicitly distinguish between acute high-dose experimental exposures and chronic low-level real-world intakes, noting that the latter may pose lower or negligible risk. To conceptually integrate the dose-dependent duality of chrysanthemum constituents and highlight current evidence gaps across exposure levels, we present a conceptual dose-response model in Table 1. Nevertheless, vulnerable populations (e.g., pregnant women) could be more sensitive, and cumulative exposure from multiple sources should be considered. Future dose-response studies using physiologically relevant concentrations and appropriate developmental windows are urgently needed.

**TABLE 1:** Conceptual dose-response model for neural tube development.

Dose	Biological effects	Key mechanisms	Effect on neural tube development	Evidence and knowledge gaps
Low (e.g., dietary/herbal tea exposure)	Anti-inflammatory, antioxidant, cytoprotective	NF-κB inhibition, Nrf2 activation, ROS reduction, anti-inflammatory cytokine suppression (TNF-α, IL-6, IL-1β)	Protection Reduced risk of NTDs via mitigation of maternal inflammation/oxidative stress	Evidence: *In vitro* anti-inflammatory and animal oxidative stress models Gap: No neurulation/human PK data
Moderate (e.g., supra-dietary, sub-toxic)	Mixed effects- mild pro-oxidant, adaptive stress response	Partial NF-κB activation, modest ROS increase, upregulation of antioxidant enzymes	Ambiguous May be protective or mildly disruptive depending on duration and fetal susceptibility	Evidence: Zebrafish show dose-dependent transition; some *in vitro* studies Gap: No data on neural tube–specific threshold
High (e.g., experimental toxicology doses)	Pro-oxidant, pro-apoptotic, neurotoxic	ROS overproduction, caspase-3 activation, PCP suppression (Vangl2, Celsr1), cytoskeletal/adhesion disruption, Wnt/Shh/Notch crosstalk	Disruption Elevated NTD risk (neural fold closure failure, altered neuroepithelial proliferation)	Evidence: High-dose pyrethroid studies in rodents; *in vitro* neurotoxicity Gap: No neurulation data; placental/fetal unknown

Abbreviations: ROS, reactive oxygen species; NF-κB, nuclear factor kappa B; Nrf2, nuclear factor erythroid 2-related factor 2; TNF-α, tumor necrosis factor alpha; IL-6, interleukin-6; IL-1β, interleukin-1, beta; NTDs, neural tube defects; PCP, planar cell polarity; PK, pharmacokinetics.

Low dose refers to typical dietary or herbal tea consumption; moderate dose represents supra-dietary but subtoxic levels; high dose corresponds to concentrations commonly used in experimental toxicology studies. Direct evidence for chrysanthemum extracts in neurulation-specific models is currently lacking across all dose ranges. Unless otherwise indicated, all cited evidence is derived from non-neurulation systems.

Taken together, although direct pharmacokinetic data on chrysanthemum-derived compounds (pyrethrins and flavonoids) during pregnancy are lacking, existing evidence indicates that flavonoids readily cross the placenta and may pose fetal risks at high doses, while pyrethrins exert multi-organ toxicity primarily through oxidative stress-mediated pathways in various experimental models. However, most toxicological studies use concentrations far exceeding typical dietary or herbal tea exposure, suggesting that real-world risks may be low for the general population. Nevertheless, vulnerable groups such as pregnant women could be more sensitive, and cumulative exposure from multiple sources warrants attention. Given that oxidative stress and dysregulated apoptosis are well-established contributors to NTDs, it is theoretically plausible that pyrethrin exposure during critical windows of neurulation may interfere with normal neural tube closure.

### 4.8 Dualistic and context-dependent biological effects of chrysanthemum

The preceding sections have detailed multiple mechanisms, here we synthesize these findings into a dualistic framework. Chrysanthemum exhibits a context-dependent duality in its biological effects, precluding its classification as uniformly beneficial or adverse. Its optimal application therefore requires the targeted selection of specific extracts based on defined pharmacological objectives.

Chrysanthemum and its bioactive constituents are widely recognized for promoting human health and supporting neural tube development. Key beneficial mechanisms include: (1) Anti-inflammatory activity via chlorogenic acid, eriodictyol-7-O-β-D-glucoside (Eri-7-Glc), and hanfangchin A through distinct molecular pathways. (2) NTDs protective potential of Eri-7-Glc by upregulating SOD and inhibiting VCAM-1 signaling. (3) Hepatoprotection via antioxidant and anti-apoptotic effects (e.g., BZE). Antioxidative and free-radical scavenging by yellow pigments and cumambrin A. Furthermore, CBME exerts significant neuroprotective effects in SH-SY5Y cells by suppressing apoptotic pathways, while CRE demonstrates clear neuroprotective properties in dopaminergic neuron models (Moon et al., 2026).

**FIGURE 1:**
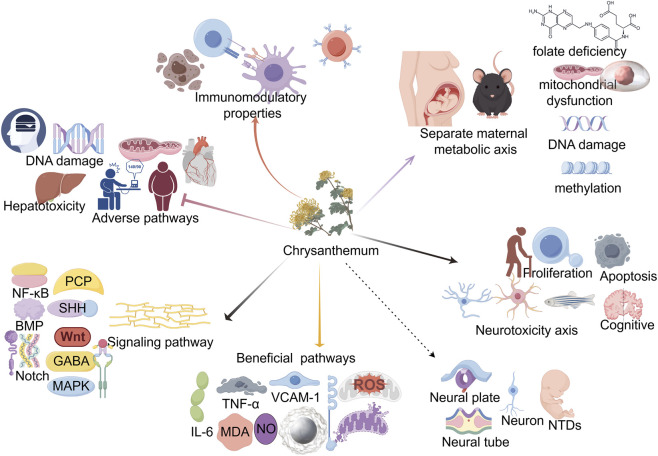
Proposed mechanistic model of chrysanthemum bioactives in NTD pathogenesis. Chrysanthemum-derived compounds exert immunomodulatory effects (center) while also activating adverse signaling pathways (left: PCP, NF-κB, SHH, BMP, Wnt, GABAA, Notch, MAPK, and inflammatory/oxidative mediators). These converge on maternal metabolic dysfunction (folate deficiency, mitochondrial impairment), DNA damage, epigenetic alterations, and aberrant proliferation/apoptosis, ultimately leading to neurotoxicity and NTDs. The model illustrates the dose-dependent duality of chrysanthemum in neural tube development.

However, plants within the Compositae family, including chrysanthemum, also exhibit notable adverse potential. They can cause allergic contact dermatitis and, during critical developmental periods, impair motor coordination and cognition. Natural pyrethrins induce DNA damage, hepatotoxicity, and neurodevelopmental disruptions, which are linked to NTDs and PD. Chrysanthemum polyphenols exhibit anti-nutritional effects by chelating iron, potentially increasing anemia risk. At high doses, certain polyphenols cause hepatotoxicity, nephrotoxicity, and genotoxicity. Quercetin can disrupt catechol-estrogen homeostasis and promote carcinogenesis, while caffeic acid induces gastric and renal tumors in rodents. Chrysanthemum flavonoids also show anti-thyroid activity, which may contribute to endemic goiter in high-consumption populations. This context-dependent duality underscores that optimal use of chrysanthemum requires tailored selection of specific extracts based on defined pharmacological goals (Figure 1).

## 5 Conclusion and perspectives

**FIGURE 2:**
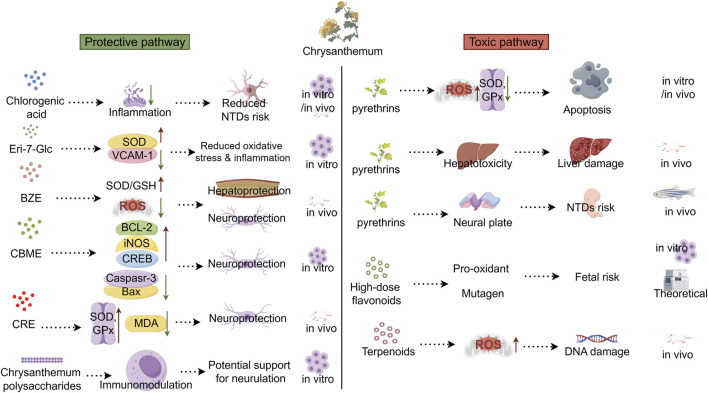
Proposed dual mechanisms of chrysanthemum constituents in neural tube development. Left indicate protective effects, right indicate adverse/toxic effects. Dashed arrows represent hypothesized links, not proven causal relationships. Evidence levels are indicated as [*in vitro*], [*in vivo*], or [Theoretical]. Solid lines (if any) indicate established mechanisms. Abbreviations: Eri-7-Glc, Eriodictyol-7-O-β-D-glucoside; BZE, Chrysanthemum boreale; CBME, Chrysanthemum boreale Makino Extract; CRE, Chrysanthemum zawadskii extract; SOD, Superoxide dismutase; GPx, Glutathione peroxidase; MDA, Malondialdehyde; ROS, Reactive oxygen species; NTDs, neural tube defects.

Chrysanthemum exhibits a context-dependent duality in neural tube development. On one hand, compounds such as chlorogenic acid, eriodictyol-7-O-β-D-glucoside, and flavonoids exert anti-inflammatory, antioxidant, immunomodulatory, and neuroprotective effects via NF-κB, Nrf2/HO-1, AMPK/GSK3β, PI3K/Akt, MAPK, and Wnt/β-catenin pathways. On the other hand, pyrethrins and high-dose polyphenols act as pro-oxidants, mutagens, and endocrine disruptors, causing DNA damage, hepatotoxicity, and developmental neurotoxicity, with flavonoids crossing the placenta and raising fetal safety concerns, suggesting a concentration-dependent double-edged sword: low doses may support neurodevelopment, while high doses may disrupt progenitor cell balance. Modulation of GABA, Shh, BMP, Wnt, Notch, and PCP pathways offers plausible hypotheses but lacks direct evidence in neurulation models. A visual summary of these dual pathways is presented in Figure 2.

Several critical gaps persist such as the absence of direct evidence in mammalian embryos, the lack of pregnancy pharmacokinetics and placental transfer data, uncharacterized dose-response relationships across developmental windows, and limited species comparisons. Future priorities should include direct testing in mammalian neurulation models, placental perfusion and pregnancy pharmacokinetic studies, dose–response and window-of-susceptibility analysis, species- and extract-specific profiling, and mechanistic dissection of dual pathways. In conclusion, although chrysanthemum exhibits potential, caution is warranted. A balanced, evidence-based approach informed by developmental toxicology and pharmacokinetics is necessary prior to any recommendation regarding its use during pregnancy or for the prevention of NTDs. This review offers a roadmap for future investigations aimed at establishing whether chrysanthemum can be safely exploited for neurodevelopmental health.

## Glossary

**AD**: Alzheimer’s disease
**AP**: acetaminophen
**AST**: Aspartate aminotransferase
**ALT**: Alanine aminotransferase
**ATP**: Adenosine triphosphate
**BZE**: Chrysanthemum boreale
**Bcl-2**: b-cell lymphoma-2
**BMI**: Body mass index
**CAT**: catalase
**CBME**: Chrysanthemum boreale Makino Extract
**Chr2**: Chromosome 2
**COX-2**: Cyclooxygenase-2
**CA**: Chrysanthemulide A
**CRE**: Chrysanthemum zawadskii extract
**CREB**: cAMP-response element binding
**CVA**: Cough Variant Asthma
**CME**: Chrysanthemum methanol extract
**CIC**: Circulating immune complexes
**CIE**: Chrysanthemum indicum extract
**CISCFE**: Chrysanthemum indicum Linné extract obtained with supercritical carbon dioxide fluid
**DR5**: Death receptor 5
**EEG**: Electroencephalography
**EEAP**: Ethyl acetate extract of chrysanthemum
**ET-1**: Endothelin-1
**Eri-7-Glc**: Eriodictyol-7-O-β-D-glucoside
**FOLR1**: Folate Receptor 1
**GFP**: Green Fluorescent Protein
**GPX**: Glutathione peroxidase
**GSH**: Glutathione
**H2O2**: Hydrogen peroxide
**Hcy**: Homocysteine
**HO-1**: Heme oxygenase 1
**IFN-r**: Interferon gamma
**IL-2**: Interleukin-2
IL-1β: Interleukin-1beta
**iNOS**: inducible Nitric oxide synthase
**JNK**: c-Jun N-terminal kinase
**LPS**: Lipopolysaccharide
**MAPKs**: Mitogen-activated protein kinases
**MDA**: Malondialdehyde
**NTDs**: Neural tube defects
**NO**: Nitric oxide
**NOS**: NO synthase
**NADPH**: Nicotinamide Adenine Dinucleotide Phosphate
**NF-κB**: Nuclear factor-kappaB
**PGE2**: Prostaglandin E2
**PD**: Parkinson’s disease
**p-AMPK**: Phosphorylated AMP-activated protein kinase
**p-GSK3β**: phosphorylated Glycogen Synthase Kinase 3β
**PI3K-Akt**: Phosphatidylinositol 3-kinase/Protein Kinase B signaling pathway
**PPARγ**: Peroxisome Proliferator-Activated Receptor gamma
**PCP**: Planar cell polarity
**ROS**: Reactive oxygen species
**SAM**: S-adenosylmethionine
**Shh**: Sonic Hedgehog
**SHR**: spontaneously hypertensive rats
**SOD**: Superoxide dismutase
**TNF-α**: Tumor Necrosis Factor-α
**TLR4**: Toll-like Receptor 4
**TPO**: Thyroid peroxidase
**VCAM-1**: Vascular Cell adhesion Molecule-1
**VO**: Volatile oil
